# 1ɑ,25‐Dihydroxyvitamin D_3_ promotes osteogenesis by down‐regulating FGF23 in diabetic mice

**DOI:** 10.1111/jcmm.16384

**Published:** 2021-02-20

**Authors:** Wenqiong Luo, Yixuan Jiang, Zumu Yi, Yingying Wu, Ping Gong, Yi Xiong

**Affiliations:** ^1^ State Key Laboratory of Oral Diseases Sichuan University Chengdu China; ^2^ National Clinical Research Center for Oral Diseases Sichuan University Chengdu China; ^3^ Department of oral Implantology West China Hospital of Stomatology Sichuan University Chengdu China

**Keywords:** 1ɑ,25‐Dihydroxyvitamin D3(1,25D), diabetes mellitus, fibroblast growth factor 23 (FGF23), osteogenesis, transcription factor Forkhead Box O1 (FOXO1)

## Abstract

1ɑ,25‐dihydroxyvitamin D3 (1,25D) and fibroblast growth factor 23 (FGF23) play important roles in bone metabolism through mutual regulation. However, the underlying mechanism between 1,25D and FGF23 in diabetes‐induced bone metabolism disorders has not yet been elucidated. In this study, we investigated the effect of 1,25D on FGF23 under diabetic condition both in vitro and in vivo. The results showed that 1,25D down‐regulated the expression of FGF23 in osteoblast significantly though a dose‐dependent manner in vitro within high glucose environment. Western blot and immunofluorescence analysis indicated that 1,25D activated PI3K/Akt signalling through binding to vitamin D receptor (VDR), which inhibited the phosphorylation of the transcription factor Forkhead Box O1 (FOXO1). Decreased phosphorylation of FOXO1 down‐regulated the expression Dickkopf‐1 (DKK1), a well‐known inhibitor of Wnt signalling. In addition, we observed that 1,25D remarkably ameliorated osteogenic phenotypic markers such as Ocn and Runx2 and rescued diabetes‐induced bone loss in vivo. Our results suggested that 1,25D could promote osteogenesis though down‐regulating FOXO1/FGF23 in diabetes.

## 1 INTRODUCTION

1ɑ,25‐dihydroxyvitamin D3 (1,25D) plays an important role in bone metabolism, which could reduce the risk of bone fracture in patients with diabetes mellitus. 1,25D is generated by the 1α‐hydroxylation reaction of 25‐hydroxyvitamin D3 (25D) catalyzed by mitochondrial CYP27B1.^1^ 1,25D affects its downstream target genes to regulate intestinal calcium absorption and bone metabolism though binding to its nuclear receptor vitamin D receptor (VDR).^2^ Increasing evidence has demonstrated that 1,25D could promote osteoblasts proliferation via increasing the production of osteopontin (Opn) and osteocalcin (Ocn).^3^, ^4^ 1,25D could also enhance the expression of membrane receptor LRP5 of Wnt pathway to stimulate osteoblasts differentiation. Conversely, 1,25D could promote the expression of RANKL and inhibit osteoprotegerin, thus accelerating osteoclastogenesis and bone resorption. Besides, research also showed that 1,25D down‐regulated the expression of Runx2 through BMP signalling pathway, thereby suppressing osteogenesis.^3^ The osteogenic or osteoclastic effects of 1,25D depends on appropriate level of 1,25D under certain conditions.

As a member of the FGF19 subfamily, FGF23 is an important regulatory factor of 1,25D, which is produced mainly by osteocytes and osteoblasts.^5^ FGF23 could reduce the expression of 1,25D and promote its catabolism by inhibiting mitochondrial CYP27B1 expression and activating CYP24A1 separately.^6^ 1,25D and FGF23 are tightly related to bone metabolism and the regulation of FGF23 by 1,25D varies in different cells. Study ^7^ conducted by Ichiro Kaneko et al showed that 1,25D down‐regulated the expression of Fgf23 in 3T3‐L1 differentiated adipocytes, while up‐regulated the expression of Fgf23 in UMR‐106 osteoblast‐like cells. In diabetic patients, FGF23 significantly elevated.^8^ Whether 1,25D could regulate FGF23 in diabetic patients, thus influencing bone metabolism and the underlying mechanisms remains unclear. Therefore, we aim to investigate the regulatory effect of 1,25D on Fgf23 and its role in bone metabolism disorder caused by diabetes in vitro and in vivo.

## 2 MATERIALS AND METHODS

### 2.1 Animal study

The animal experiments were approved by the Animal Research Committee of Sichuan University (Chengdu, China). Animal care was conducted followed the National Institutes of Health (NIH Publications No. 8023, revised 1978). FoxO1^fl/fl^ mice and BGLAP‐Cre mice were purchased from Jackson Laboratories (Bar Harbor, ME) and crossed for the production of FoxO1^fl/fl^Cre^±^ and FoxO1^fl/fl^Cre^‐/‐^ mice.^9^ Forty male mice (6‐week‐old) were divided into five groups (n = 8 mice per group) by simple randomization method as follow: WT mice (WT); untreated diabetic WT mice (WT‐STZ); 1,25D treated diabetic WT mice (WT‐STZ‐1,25D); untreated diabetic KO mice (KO‐STZ); 1,25D treated diabetic KO mice (KO‐STZ‐1,25D). To establish diabetes model, mice were fed with a high‐fat diet (60 kcal% fat, 20 kcal% carbohydrate, 20 kcal% protein; D12492 diet; Research Diets) for 3 weeks before the injection of streptozotocin (STZ, Sigma) at a repeated dose of 40 mg/kg.^10^ Ten days after STZ administration, mice with blood glucose levels of > 150 mg/dL than control mice were considered to be a successful model. The 1,25D treated groups received a dose of 5 ug/kg via intraperitoneal injection every other day for 4 weeks. Blood was collected though a cheek pouch puncture 24 h after the last injection. Simultaneously vertebrae were harvested.^11^

### 2.2 Micro‐CT analysis

Vertebrae were fixed and scanned by micro‐CT (Micro CT 50, SCANCO Medical AG, Switzerland) at 7μm resolution. The following indexes of bone were analysed: bone volume per total volume (BV/TV), the mean trabecular number (Tb.N) and the mean trabecular thickness (Tb. Th) and the mean trabecular separation (Tb. Sp ).

### 2.3 Enzyme‐linked immunosorbent assay (ELISA)

Blood serum from mice was harvested and centrifuged for 10 minutes at 1000 g. Serum concentrations of FGF23, P1NP and CTX were detected using ELISA kits (Fountain Hills, AZ) according to the manufacturer's instructions. Serum calcium and phosphate were detected by multichannel autoanalyser (Hitachi 717, Boehringer Mannheim).

### 2.4 Cell culture

Calvaria from newborn WT and KO mice were collected for primary osteoblasts culture using direct explants culture method.^12^ Cells were cultured in α‐MEM medium supplemented with 10% foetal bovine serum (FBS, Gibco), 100 U/mL penicillin and 100 mg/mL streptomycin sulphate. Osteoblasts in their third passage were reseeded and incubated in normal glucose (5.5 mm) and high glucose (22 mm) with or without 1,25D. PI3K inhibitor LY294002 (20 μm) and Akt inhibitor MK‐2206 (1 μm) were used to inhibit PI3K/Akt pathway. VDR Crispr/Cas9 KO Plasmid (Santa Cruz) was applied to knockout VDR in osteoblasts according to manufacturer's instruction, which has been previously described.^13^ Lentiviral vector carrying FoxO1 gene was used overexpress FoxO1 according to the instructions of the manufacturer. The effect of plasmid transfection and lentivirus transfection was verified by western blot. Concentration of FGF23 in supernatant of Osteoblasts treated with lentivirus vector and 1,25D was determined by ELISA.

### 2.5 Real‐Time PCR and Western blotting Analysis

Total RNAs were extracted using TRIzol Regent (Invitrogen). mRNAs were reverse transcribed into cDNA using PrimeScript™ RT reagent Kit with gDNA Eraser (Takara Bio, Japan) in a 10 μL reaction volume. Primers sequences were listed in Table 1. The relative gene expression was calculated by normalizing to endogenous housekeeping gene *Gapdh* using 2^−△△Ct^ method with ABI 7300 real‐time PCR system (Applied Biosystems) and SYBR Premix Ex Taq (Takara Bio).

**TABLE 1:** Primers sequences for RT‐qPCR

Gene	Forward 5'→3'	Reverse 5'→3'
*Fgf23*	GGCTGCTGGCTTCTAAGTGTG	TTCCGTGACCGGTAAGTATTG
*Ocn*	GCGCTCTGTCTCTCTGACCT	ACCTTATTGCCCTCCTGCTT
*Runx2*	GGCGTCAAACAGCCTCTTCA	GCTCACGTCGCTCATCTTGC
*Cathepsin K*	CCAGTGGGAGCTATGGAAGA	TCTGCTGCACGTATTGGAAG
*Sfrp1*	CCCTCCAAGGCTTGAGTAAAAG	AGCACATGCATAGGCGGTGTA
*Sfrp4*	TGGAGCCACCCTTACAGGAT	GCAAGTGGTATGTGGCCTTCTG
*Dkk1*	ACTCAAATGGCTTTGGTAATATGG	ATAATCTCTTCTGAATTCTGCCCA

For western blot, cell proteins were obtained using KeyGEN Whole Cell Lysis Assay (KeyGEN) according to the manufacturer's instructions. Protein extracts were separated on a 10% SDS‐PAGE gels and transferred on polyvinylidene difluoride membranes (Millipore Corp), which were then blocked and probed at 4°C overnight with primary antibodies: rabbit polyclonal anti‐VDR (1:100, Abcam), rabbit polyclonal anti‐pAkt (1:500, Cell Signaling Technology), rabbit monoclonal anti‐Akt (1:500, Cell Signaling Technology), rabbit monoclonal anti‐pFOXO1(1:100, Santa Cruz), rabbit polyclonal anti‐FOXO1(1:100, Santa Cruz), rabbit polyclonal anti‐DKK1 antibody(1:1000, Abcam). Rabbit monoclonal anti‐GAPDH (1:1000, Cell Signaling) was applied as loading controls. After incubated with HRP‐conjugated secondary antibody (1:1000), immunoreactive bands were detected using an ECL kit (Millipore).

### 2.6 Immunofluorescence and immunohistochemistry

Cells were seeded on coverslips in a 48‐well plate at a density of 10 000 cells/well. After 72 hours, the slips were gently rinsed with PBS and fixed with 4% paraformaldehyde for 30 minutes, then permeabilized with 2% Triton X‐100 for 5 minutes and blocked with 5% bovine serum albumin (Sigma) for 30 minutes. Cells were incubated with primary antibody FOXO1(1:50, Santa Cruz Biotechnology) at 4℃ overnight. Slips were incubated with fluorescein isothiocyanate‐conjugated secondary antibodies (1:50; Zsjq Bio Co., Beijing, China) for 1 hours at room temperature. Nuclei were stained with DAPI (1:1000; Beyotime, Shanghai). Fluorescent images were collected by fluorescent microscopy (Olympus, Tokyo, Japan).

For immunohistochemical staining, femurs were harvested and firstly fixed in 4% paraformaldehyde for 2 days. Washed femurs were subsequently put into 10% EDTA‐buffered solution until complete demineralization and then dehydrated with graded alcohol, vitrified in xylene before embedded in paraffin. Bone sections were cut into 5 μm thickness longitudinally, quenched in 3% H_2_O_2_ for 5 minutes and unmasked through submerged in boiling citrate buffer (pH 6.0) three times for 8 minutes each. Then sections were washed and blocked in 5% goat serum before incubated with Runx2 (Abcam), DKK1 (Abcam), FGF23 (Abcam) antibodies at 4℃ overnight. Sections were rewarmed at room temperature before incubated with secondary antibodies. Stained sections were optimized with 3,3′‐diaminobenzidine (DAB) and counterstained with haematoxylin. TRAP staining kit (Sigma) was used to observe bone resorption. TRAP‐positive multinucleated cells with three or more nuclei were scored. All stained sections were visualized under a microscope using NIS‐Elements software (Nikon Eclipse 80i, Nikon).

### 2.7 Statistical analysis

All statistical tests were performed with SPSS 20.0 (SPSS, Inc). Unpaired two‐tailed Student's *t* test was used between two groups and one‐way ANOVA followed by Tukey's test was used for multiple comparisons. The results were given as means ± SD *P* <.05 was considered to be statistically significant.

## 3 RESULTS

### 3.1 1,25D down‐regulates *Fgf*23 expression by activating PI3K/Akt signalling pathway

**FIGURE 1:**
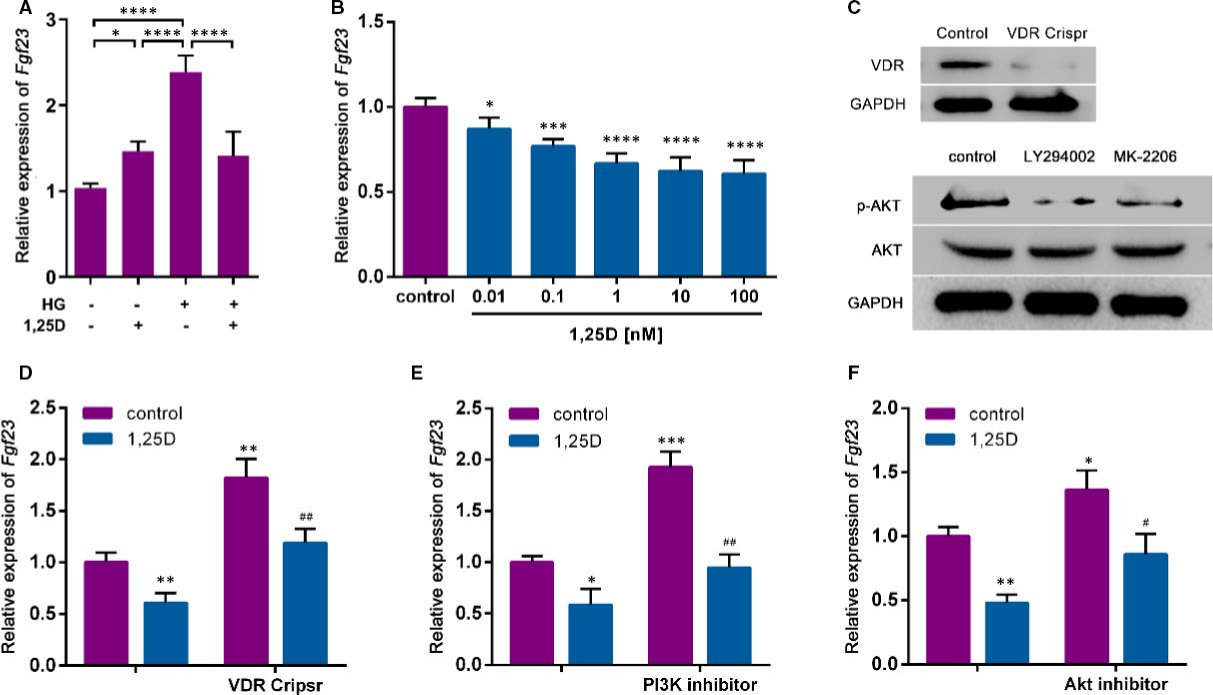
1,25D down‐regulates *Fgf23* gene expression by activating PI3K/Akt signalling in treated osteoblasts. A: mRNA level of *Fgf23* of osteoblasts treated in normal and high glucose media with or without 1,25D (10nM). B: *Fgf23* expression at different 1,25D concentration. C: Protein levels of VDR, p‐Akt and Akt in treated osteoblasts tested by Western blot analysis. D‐F: mRNA level of *Fgf23* in osteoblasts with VDR knockout, PI3K inhibitor LY294002 or PKB/Akt inhibitor MK‐2206. Gene expression was normalized to *Gapdh* as a housekeeping gene, and the results are shown as mean ± SD, n = 4 specimens/group. *P <.05; **P <.01, ***P <.001, for control vs. others; # *P* <.05, ## *P* <.01, for 1,25D vs. 1,25D combined with VDR Crispr or PI3K inhibitor or Akt inhibitor; VDR Cripsr represents VDR knockout

To evaluate the effect of 1,25D on *Fgf*23 expression, real‐time qPCR was conducted. The expression of *Fgf*23 was significantly increased in a high glucose environment. In normal medium, 1,25D promoted the expression of *Fgf*23, while in high glucose medium 1,25D inhibited the expression of *Fgf*23 (Figure 1A). Furthermore, as shown in Figure 1B. 1,25D significantly down‐regulated *Fgf*23 expression in a dose‐dependent way. 10 nm, 1,25D showed remarkable effect on the expression of *Fgf*23 tended to be stable, which has been applied in following experiments. To further explore the underlying mechanism, Crispr/Cas9 KO plasmid was applied to knockout VDR, which was verified by western blot analysis (Figure 1C). The absence of VDR restored the inhibitory effect of 1,25D on *Fgf*23 (Figure 1D). Previous studies have shown that the PI3K/Akt pathway could play an important role in cell growth, proliferation, survival and metabolism.^14^ Therefore, PI3K inhibitor LY294002 and Akt inhibitor MK‐2206 were also applied to block PI3K/Akt signalling pathway in osteoblasts (Figure 1C). As shown in Figure 1E, *Fgf*23 mRNA levels increased with the application of PI3K inhibitor, while decreased with 1,25D. Similar results were found when Akt inhibitor was applied (Figure 1F). Taken together, these results suggested that 1,25D down‐regulated *Fgf*23 expression by activating PI3K/Akt signalling pathway.

### 3.2 The Effect of 1,25D on FGF23 is mediated via inactivation of FOXO1

**FIGURE 2:**
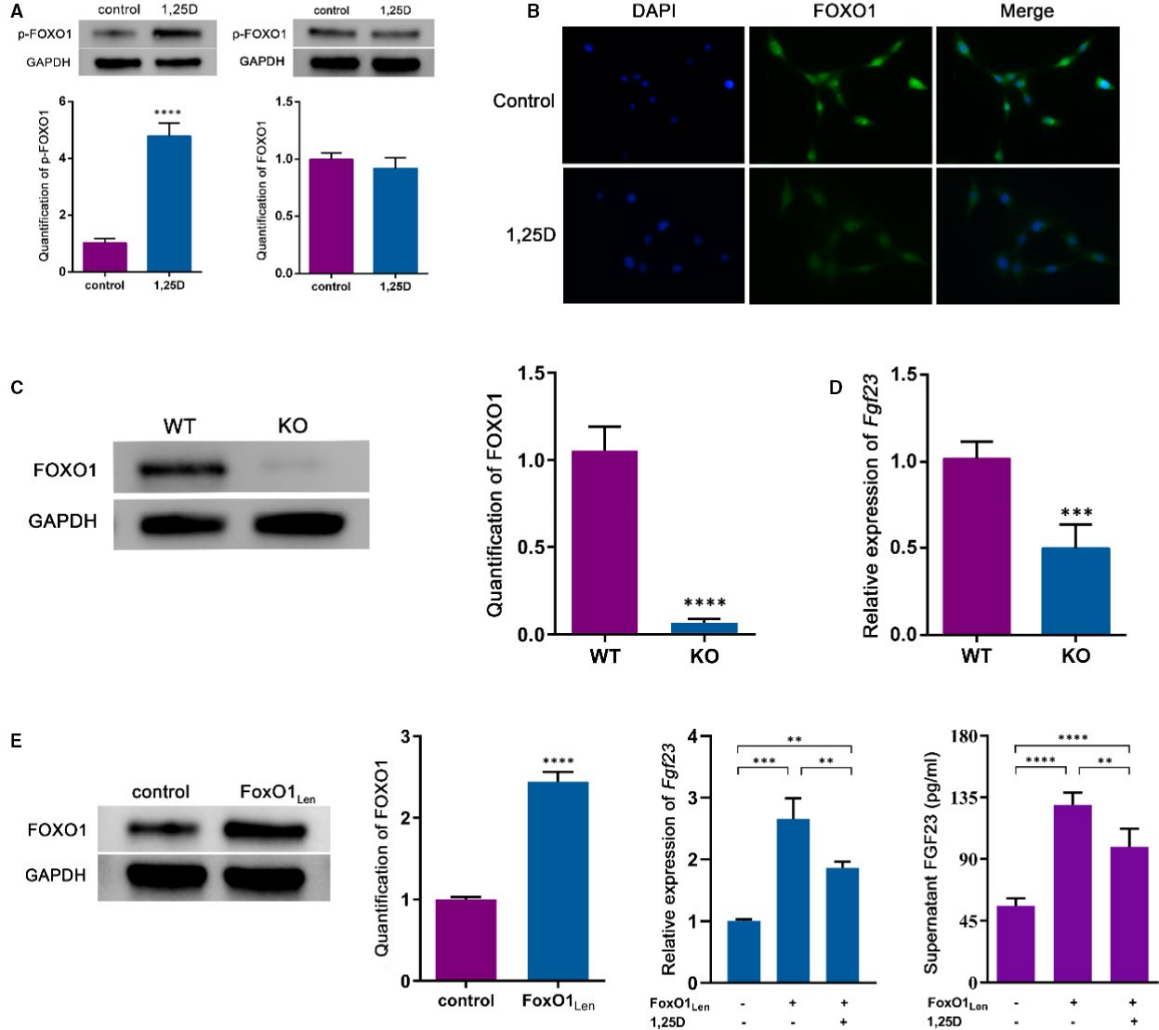
The effect of 1,25D on *Fgf23* is mediated by inactivation of FOXO1. A: Western blot analysed the quantification of FOXO1 and p‐FOXO1 in control and 1,25D treated osteoblasts. B: FOXO1 of osteoblasts was detected by immunofluorescent staining, scale bar = 20μm. C: FoxO1 knockout in osteoblasts was proved by western blot. D: the mRNA level of *Fgf23* when FoxO1 was knockout was detected by RT‐PCR. E: The result of lentivirus transfection in FoxO1 knocked osteoblasts was verified by western blot. Osteoblasts were infected with lentivirus (50 MOI) or control vector. 24 hours later, cells were treatment with 1,25D (10 nm), and then the mRNA level and supernatant concentration of FGF23 was detected. ***P <.001, for KO vs. WT; **** *P* <.0001. KO represents FoxO1 knockout, WT represents control

As one of the downstream targets, FOXO1 can be phosphorylated by serine‐threonine kinase Akt such as Ser256.^15^ Thus, we hypothesized that FOXO1 might act as a mediator in the effect of 1,25D on FGF23 expression. Western blot analysis showed that 1,25D treatment led to the phosphorylation of FOXO1 (p‐FOXO1) (Figure 2A). In addition, 1,25D decreased FOXO1 nuclear location as the cell immunofluorescent staining displayed (Figure 2B), indicating that 1,25D might restrain the shuttle of FOXO1 from cytoplasm to nucleus and promoted FOXO1 nuclear exclusion. To further validate that FOXO1 might be responsible for *Fgf*23 gene transcription, osteoblasts from conditional FoxO1 knockout mice and lentivirus vector carrying FoxO1 gene transduced Osteoblasts were applied in the study (Figure 2C,2E). When compared with WT group, the mRNA level of *Fgf*23 in KO group reduced remarkably (Figure 2D). Overexpression of FoxO1 up‐regulated the expression of *Fgf*23 whereas alleviated by the treatment of 1,25D. We also detected FGF23 level in supernatant, which was consistent with the trend of mRNA level (Figure 2E). Collectively, these findings confirmed that the effect of 1,25D on FGF23 expression might be mediated via the inactivation of FOXO1.

### 3.3 1,25D promoted osteogenesis via inactivation of FOXO1 in diabetic mice

**FIGURE 3:**
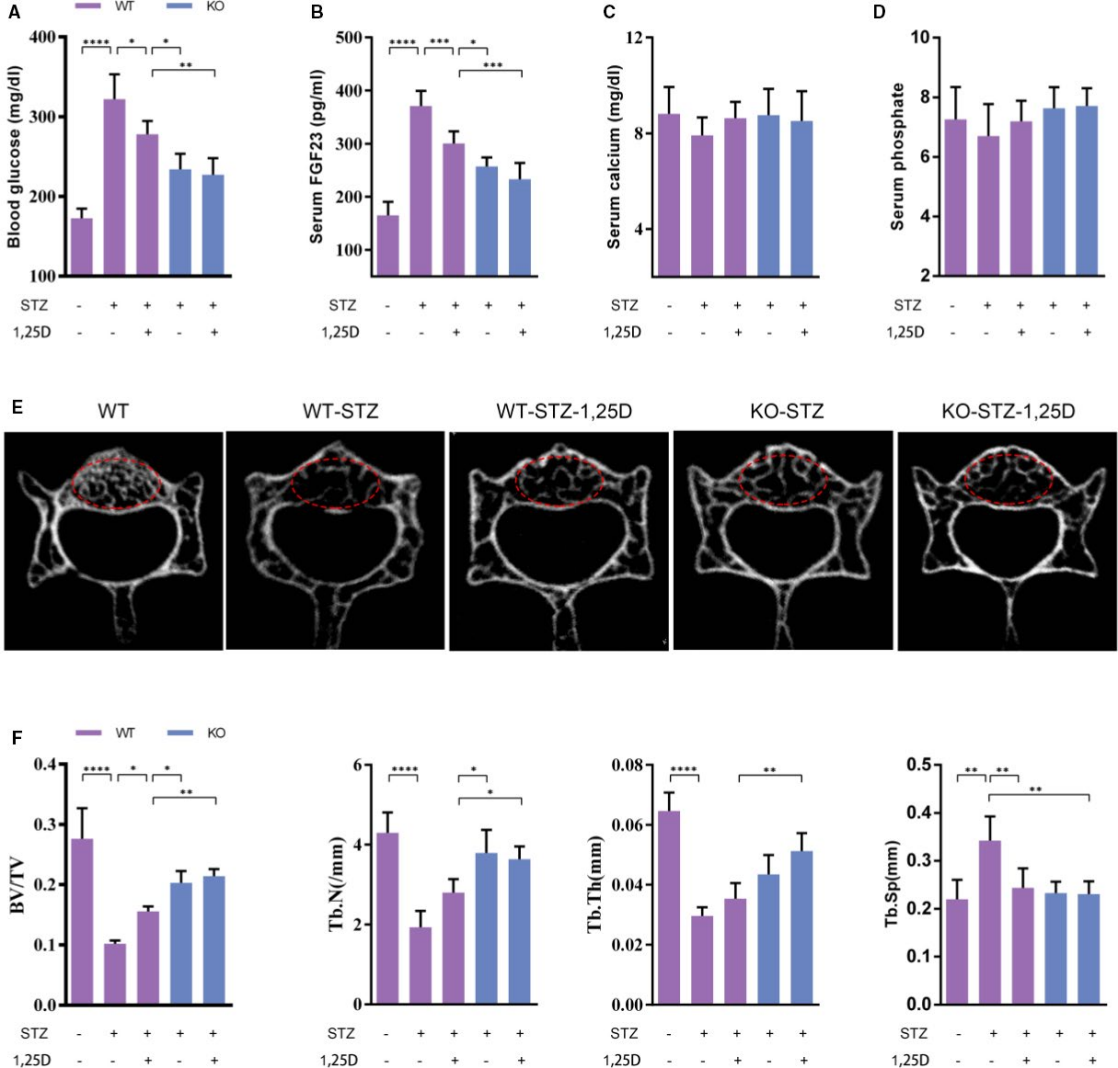
FoxO1_OB_^‐/‐^ and 1,25D treatment promote osteogenesis in mice. The level of blood glucose (A), Serum FGF23 (B), Serum calcium (C) and phosphate (D). E: Micro‐CT scanning result of vertebrae. F: Result of BV/TV, Tb.N, Tb. Th and Tb. Sp Results are shown as mean ± SD; n = 4 pecimens/group. *P <.05, **P <.01, ***P <.001, ****P < 0,0001. WT: wild type; WT‐STZ: untreated diabetic WT mice; WT‐STZ‐1,25D: 1,25D treated diabetic WT mice; KO: mice with conditional FoxO1 knockout in osteoblasts; KO‐STZ: untreated diabetic KO mice; KO‐STZ‐1,25D: 1,25D treated diabetic KO mice

**FIGURE 4:**
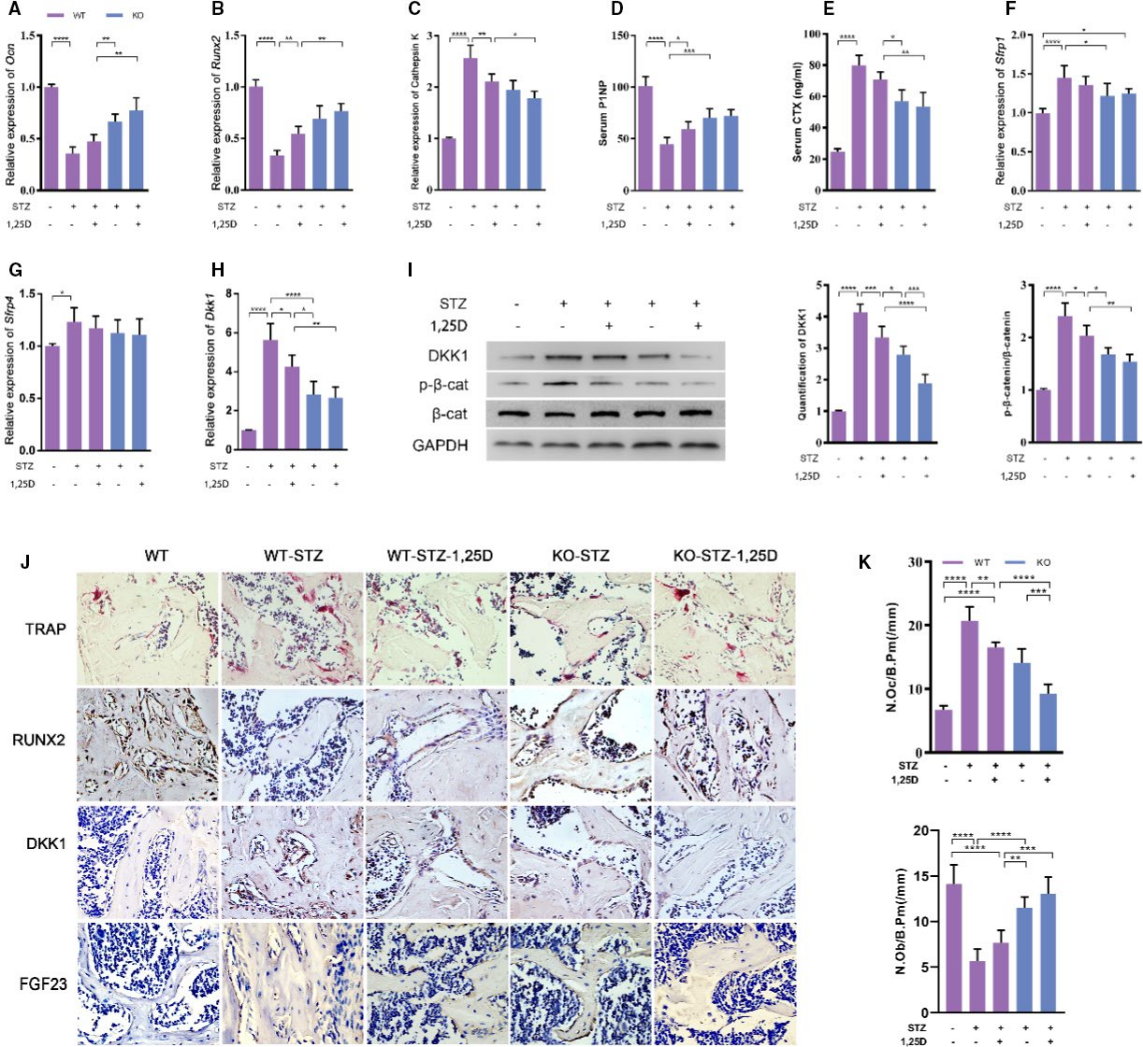
FOXO1 might mediate 1,25D‐stimulated β‐catenin expression. mRNA expression of osteogenic gene *Ocn* (A), *Runx2* (B), and osteoclastic gene *Cathepsin K* (C). Serum osteogenic protein P1NP (D) and osteoclast CTX(E) were detected by ELISA (n = 5 specimens/group). F‐H: The inhibit gene to β‐catenin: *Sfrp1* (F), *Sfrp4* (E) and *Dkk1* (H) was in accordance with the change of β‐catenin in protein level (I). J: Immunochemical staining of TRAP, Runx2, DKK1 and FGF23, scale bar = 50µm. K: The number of osteoclasts and osteoblasts was measured to assess the osteoclast activity. Data are presented as mean ± standard deviation (SD), n = 3 specimens/group. *P <.05, **P <.01, *** *P* <.001, ****P <.0001. WT: wild type; WT‐STZ: untreated diabetic WT mice; WT‐STZ‐1,25D: 1,25D treated diabetic WT mice; KO: mice with conditional FoxO1 knockout in osteoblasts; KO‐STZ: untreated diabetic KO mice; KO‐STZ‐1,25D: 1,25D treated diabetic KO mice

Evidence has shown that diabetes mellitus is always accompanied with hypocalcaemia and hypophosphatemia, which could be ameliorated by 1,25D.^16^ In this study, we established type 2 diabetic mice model to clarify the effect of 1,25D on bone microarchitecture and its underlying mechanism. As shown in Figure 3A, WT‐STZ mice had a higher blood glucose level than WT mice, while both 1,25D treatment and FoxO1 knockout showed positive effects on glucose level. We then detected serum FGF23 level. Results similar variations as blood glucose (Figure 3B). In brief, serum FGF23 increased significantly to a concentration of 371.2 pg/mL in WT‐STZ mice and decreased to 300.8 pg/mL with the treatment of 1,25D (*P* <.05). Besides, FoxO1 knockout reduced serum FGF23 level in KO‐STZ mice and KO‐STZ‐1,25D mice, with significant difference compared with WT‐STZ‐1,25D mice (*P* <.05). Of note, there was no statistical difference of serum concentration of FGF23 between KO‐STZ‐1,25D mice and KO‐STZ mice (*P* >.05) (Figure 3B), suggesting that FOXO1 was involved in the effect of 1,25D on FGF23. The results of immunohistochemistry showed the similar variation (Figure 4J). Though serum calcium and phosphate slightly decreased in WT‐STZ mice, there was no significant difference among the five groups (Figure 3C‐D).

Micro‐CT analysis revealed that WT‐STZ group showed obvious bone loss when compared with WT, with 63.1%, 55.0% and 54.2% reduction of BV/TV, Tb.N and Tb. Th, respectively (Figure 3F). 1,25D ameliorated diabetes‐induced bone loss in WT‐STZ‐1,25D mice to some extent, with statistical difference in BV/TV parameter (*P* <.05). KO‐STZ and KO‐STZ‐1,25D mice displayed no significant difference in all microarchitecture parameters (*P* >.05), while osteogenesis was better in these two groups when compared with WT‐STZ‐1,25D mice, indicating that FOXO1 might be a mediator during the regulatory effect of 1,25D on osteogenesis.

Then we detected the expression of osteogenic markers such as *Ocn, Runx2* and osteoclastic marker *Cathepsin K* in femur. Real‐time qPCR analysis displayed that the expression of *Ocn* and *Runx2* decreased remarkably in WT‐STZ mice, while *Cathepsin K* expression increased sharply. 1,25D treatment or FoxO1 knockout rescued the inhibitory effect of diabetes on osteoblastic markers to a large extent, and down‐regulated the expression of *Cathepsin K* gene (*P* <.05). Still, we found little difference of *Ocn*, *Runx2 and Cathepsin K* level between KO‐STZ mice and KO‐STZ‐1,25D mice (*P* >.05) (Figure 4A‐C). Furthermore, two typical serum markers reflecting bone formation and resorption, P1NP and CTX respectively, were assayed. Results showed that the P1NP level was lower in the WT‐STZ group while it was rescued partially by 1,25D and FoxO1 knockout. The expression of CTX showed converse changes (Figure 4D‐E). Similar results were shown in TRAP staining and immunohistochemical analysis for Runx2 (Figure 4J). TRAP staining revealed that the number of osteoclasts was increased by STZ treatment (*P* <.0001), whereas significantly decreased by 1,25D (*P* <.01) or FoxO1 knockout (*P* <.01). Although the number of osteoclasts in KO group was less than that in 1,25D treatment alone, there was no statistical difference between them (*P* >.05). The KO group treated with 1,25D has the least number of osteoclasts(*P* <.0001). The number of osteoblasts was also counted, showing the opposite trend with osteoclasts. These findings suggested that 1,25D promoted osteogenesis via inactivation of FOXO1 in diabetic mice.

### 3.4 Contribution of FOXO1/DKK1 Inactivation to WNT/β‐Catenin pathway activation

Wnt/β‐catenin pathway is one of the most important pathways for bone formation.^17^ First, we detected the expression of p‐β‐catenin/β‐catenin (p‐β‐cat/β‐cat), which reflected Wnt/β‐catenin activity. As shown in Figure 4I, p‐β‐catenin/β‐cat ratio in WT‐STZ mice was 4 times higher than that in WT mice. Both 1,25D treatment and FoxO1 knockout could decrease the ratio, implying that they could activate β‐catenin signalling (*P* <.05). No statistical difference was found between KO‐STZ and KO‐STZ‐1,25D mice, suggesting that FOXO1 might be the main mediator in the effect of 1,25D on β‐catenin expression. To explore the possible targets of FOXO1, we assessed the expression of *Sfrp1, Sfrp4* and *Dkk1*, which were known as inhibitors of β‐catenin activity. We found that *Dkk1* was 5.6‐fold higher in WT‐STZ mice than that in WT mice. Diabetes could induce the expression of *Sfrp1* and *Sfrp4*, but FoxO1 knockout slightly reduced *Sfrp1* level. There was no significant influence of 1,25D treatment on prohibiting the expression of *Sfrp1* and *Sfrp4* (Figure 4F‐G). Additionally, 1,25D could restrained mRNA level of *Dkk1* according to real time PCR (*P* <.05). *Dkk1* mRNA and protein levels in KO‐STZ and KO‐STZ‐1,25D mice were much lower than those in WT‐STZ mice, which indicated that FOXO1 inactivation resulted in the *Dkk1* inhibition, at least in part (Figure 4H‐I). Besides, it was observed that the change of DKK1 was in accordance with the change of p‐β‐catenin in protein level. Similar results were found in immunohistochemical analysis for DKK1 (Figure 4J). These data demonstrated that 1,25D might play a critical role in osteogenesis via FOXO1/DKK1 inactivation, thus activating β‐catenin signalling pathway.

## 4 DISCUSSION

1,25D is regarded as a key factor in bone metabolism, which can promote bone formation under high glucose environment.^9^ This study revealed that 1,25D down‐regulated FGF23 expression through inactivation of FOXO1 via VDR/PI3K/Akt pathway, thereby promoting osteogenesis in high‐glucose environment.

The PI3K/Akt signalling pathway plays a pivotal role in organismal growth.^18^ Consistent with previous studies, our results suggested that 1,25D could activate PI3K/Akt signal by binding to VDR.^19^ As a downstream target of PI3K/Akt, FOXO1 can be directly inhibited by phosphorylation. When FOXO1 was phosphorylated, a variety of cellular processes involved were altered, such as antioxidant stress, apoptosis, autophagy and cell metabolism.^16^ Increasing evidence has demonstrated that 1,25D could promote the phosphorylation of FOXO1, leading to FOXO1 suppression.^20^, ^21^ In our work, it was observed that 1,25D could inhibit the activation of FOXO1 through VDR/PI3K/Akt signalling transduction pathway, thus inhibiting the expression of FGF23. Contrary to our results, many studies demonstrated that 1,25D could up‐regulate FGF23 expression.^11^, ^22^ The differences might be due to the fact that FOXO1 is involved in insulin resistance and cytokine‐mediated β‐cell failure in type 2 diabetes patients. High‐glucose environment could cause oxidative stress damage and accumulation of advanced glycosylation end products, thereby affecting the downstream mechanism of VDR. 1,25D was more inclined to exert antioxidant effects to inhibit the accumulation of FGF23 in high‐glucose environment rather than promote FGF23 production as it in normal physiological condition. Besides, the regulation of FGF23 by 1,25D in a systemic setting might be partially dependent on phosphorus. When the phosphorus level does not change significantly, it is insufficient to stimulate the compensatory effect of FGF23.^23^

In vivo, we found that the blood glucose level increased accompanied with increased serum FGF23 concentration, which was consistent with the previous study conducted by Antoaneta Gateva et al^24^ Also, we detected that DKK1, an inhibitor of the Wnt pathway, was significantly down‐regulated after treatment of 1,25D or FoxO1 knockout. It is known that Wnt/β‐catenin is a crucial signalling pathway in the process of bone formation. DKK1, a typical Wnt antagonist, could inhibit Wnt‐mediated tissue repair.^25^ Sungsin Jo revealed that 1,25D significantly promoted the DKK1 expression, which is necessary for the mineralization of osteoblasts.^26^ However, some studies argued that there was a negative correlation between bone mineralization density and serum DKK1 in type 2 diabetes.^27^ In this work, higher DKK1 level was observed in STZ‐induced diabetic mice, while 1,25D slowed the upward trend of DKK1. Inhibition of FOXO1 might be involved in the mechanism whereby DKK1 was down‐regulated. The underlying mechanism may be that FOXO1 competes with TCF to interact with β‐catenin, thereby inhibiting TCF transcription activity.^28^ When FOXO1 expression was prohibited, nucleus β‐catenin could be enhanced. Then, feedback regulation reduced the expression level of DKK1 and removed its inhibitory effect on the phosphorylation of β‐catenin.^29^ Carrillo‐López N et al^30^ also suggested that FGF23 could directly restrain Wnt pathway through a novel autocrine or paracrine mechanism.

In summary, our study illuminated the effects of 1,25D on the regulation of bone formation and its endocrine regulatory role in diabetes mellitus. These data demonstrated that 1,25D could regulate FGF23 expression through binding to its receptor VDR via PI3K/Akt/FOXO1 signalling pathway. In addition, the osteoprotective effect of 1,25D in diabetic mice was related to the inhibition of FOXO1 and DKK1. Our study provided a novel idea for 1,25D treatment of diabetes‐related bone disease. However, the underlying mechanism between FOXO1 and DKK1 still needs further studies.

## CONFLICT OF INTEREST

The authors declare no conflicts of interest in regards to this manuscript.

## AUTHOR CONTRIBUTION

**Wenqiong Luo:** Formal analysis (lead); Methodology (lead); Writing‐original draft (lead). **Yixuan Jiang:** Formal analysis (supporting); Methodology (supporting); Writing‐original draft (supporting). **Zumu Yi:** Data curation (equal). **Yingying Wu:** Methodology (equal); Project administration (equal); Writing‐review & editing (equal). **Ping Gong:** Conceptualization (equal); Supervision (equal). **Yi Xiong:** Conceptualization (equal); Funding acquisition (equal); Writing‐review & editing (equal).

## Data Availability

All data generated or analyzed during this study are included in this article.
